# Supplementary material for: Lung function and peak oxygen uptake in chronic obstructive pulmonary disease phenotypes with and without emphysema
Source: PLoS One. 2021 May 27;16(5):e0252386. doi: 10.1371/journal.pone.0252386 (PMC8158861; doi:10.1371/journal.pone.0252386)
Supplement: Attachment — Submitted filename: Response to Reviewers.docx [file pone.0252386.s002.docx]

Journal Requirements:

When submitting your revision, we need you to address these additional requirements.

1.) Please ensure that your manuscript meets PLOS ONE's style requirements, including those for file naming. The PLOS ONE style templates can be found at

<https://journals.plos.org/plosone/s/file?id=wjVg/PLOSOne_formatting_sample_main_body.pdf> and

<https://journals.plos.org/plosone/s/file?id=ba62/PLOSOne_formatting_sample_title_authors_affiliations.pdf>

**Response:**

**In the current version of the manuscript, corrections have been made in order to meet PLOS ONE`s style requirements.**

2.) Please include the date(s) on which you accessed the databases or records to obtain the data used in your study.

**Response:**

**In the revised version of the manuscript, this information is now included.**

**Materials and methods, page 6, line 109-110:**

***“The clinical database was accessed in the time period between the fifteenth of July and the thirtieth of August 2015.”***

Additional Editor Comments:

This is an intriguing manuscript that can be implemented enough to considered for publication in Plos One. Please follow the suggestions of the reviewer.

This retrospective study evaluating the association between the FEV1-1Zscore and VO2 peak in E and N COPD is intruging and well written, but methodologically too weak and to be suitable for publication in Plos One. The suggestions from the reviewer could implement the manuscript for a second consideration.


[Note: HTML markup is below. Please do not edit.]

Reviewers' comments:

Reviewer's Responses to Questions

**Comments to the Author**

1. Is the manuscript technically sound, and do the data support the conclusions?

The manuscript must describe a technically sound piece of scientific research with data that supports the conclusions. Experiments must have been conducted rigorously, with appropriate controls, replication, and sample sizes. The conclusions must be drawn appropriately based on the data presented.

Reviewer #1: Partly

2. Has the statistical analysis been performed appropriately and rigorously?

Reviewer #1: Yes

3. Have the authors made all data underlying the findings in their manuscript fully available?

The [PLOS Data policy](http://www.plosone.org/static/policies.action#sharing) requires authors to make all data underlying the findings described in their manuscript fully available without restriction, with rare exception (please refer to the Data Availability Statement in the manuscript PDF file). The data should be provided as part of the manuscript or its supporting information, or deposited to a public repository. For example, in addition to summary statistics, the data points behind means, medians and variance measures should be available. If there are restrictions on publicly sharing data—e.g. participant privacy or use of data from a third party—those must be specified.

Reviewer #1: Yes

4. Is the manuscript presented in an intelligible fashion and written in standard English?

PLOS ONE does not copyedit accepted manuscripts, so the language in submitted articles must be clear, correct, and unambiguous. Any typographical or grammatical errors should be corrected at revision, so please note any specific errors here.

Reviewer #1: Yes

5. Review Comments to the Author

Please use the space provided to explain your answers to the questions above. You may also include additional comments for the author, including concerns about dual publication, research ethics, or publication ethics. (Please upload your review as an attachment if it exceeds 20,000 characters)

Reviewer #1: This is a retrospective study, evaluated the association between the FEV1-1Zscore and VO2 peak in E and N COPD. The authors demonstrated that there is not a statistical difference between the association FEV1-1Zscore- VO2 and the COPD phenotypes.

The idea of this study to associate VO2 with FEV1-1zscore is very interesting and original, and the data are also interesting, but in my opinion the aim of the study is not clear.

**Response:**

**We have rephrased the following sections of the manuscript in order to clarify the aims of the study.**

**Abstract, page 3, line 31-38:**

***“Previous studies in chronic obstructive pulmonary disease (COPD) have not taken sex, age and height related variance of dynamic lung volumes into account, when reporting associations of forced expiratory lung volume in one second (FEV_1_) with peak oxygen uptake (VO_2peak_) and differences in VO_2peak_ comparing phenotypes characterized by degree emphysema. We aimed to assess the association of FEV_1Z-score_ with VO_2peak_ in COPD (n = 186) and investigate whether this association differs between emphysema (E-COPD) and non-emphysema (NE-COPD) phenotypes. Additionally, phenotype related differences in VO_2peak_ were compared using FEV_1Z-score_ and percent of predicted FEV_1_ (ppFEV_1_).***

**Introduction, page 5-6, line 99-103:**

***“We aimed to assess the association of FEV_1Z-score_ with VO_2peak_ in COPD and investigate whether this association differs comparing phenotypes with and without emphysema. Additionally, we explored the potential influence of sex, age and height related variance in FEV_1_ on both the association of FEV_1_ with VO_2peak_ and on the difference in VO_2peak_ between E-COPD and NE-COPD.”***

Reviewer #1: Authors should compare the associations of FEV1% predicted-VO2 in both groups and compare them to those of FEV1-1zscore-VO2 and see if the association is lost with the predicted ones. Then they would confirm that the weak association of FEV1predicted-VO2 depends on how the predicted ones are calculated, otherwise this work remains just descriptive and becomes just descriptive.

I think the authors should re-analyse the data comparing the correlation of FEV1%pr/VO2 versus FEV1-zscore/VO2.

**Response:**

**As suggested by the reviewer we have reanalyzed the data to compare the associations of FEV_1Z-score_ and percent of predicted FEV_1_ (ppFEV_1_) with VO_2peak_.**

**Given the different units of measurements, we have standardized ppFEV_1_ in order to compare the regression coefficients with those of FEV_1Z-score_.**

**Additionally, we have extended the analyses to compare the phenotype related difference in VO_2peak_ when adjusting for FEV_1Z-score_ and ppFEV_1_ in separate models.**

**The following sections have been revised as follows:**

**Methods, page 8, line 165-166:**

***“Corresponding analyses with standardized ppFEV_1_, calculated as (observed value – sample mean)/SD, were performed for comparison.”***

**Methods, page 9, line 168-172:**

***“Linear regression models were also used to investigate whether the difference in VO_2peak_ between E-COPD and NE-COPD is influenced by sex, age and height related variance in FEV_1_. VO_2peak_ was regressed on COPD phenotype and ppFEV_1_ and FEV_1Z-score_ in separate total sample models with adjustment for sex, age, BMI, smoking status, beta-blocker and bronchodilator treatment.”***

**Results, page 14-16, line 252-274:**

**“*Associations of standardized ppFEV_1_ with VO_2peak_ and effect modification by COPD phenotype.***

***Adjusted for COPD phenotype, sex, age, BMI, smoking status, beta-blocker and bronchodilator treatment, one unit reduction in standardized ppFEV_1_ (i.e. one SD reduction in ppFEV_1_) was, on average, associated with 2.5 (95% CI 1.8, 3.2) ml/kg/min lower VO_2peak_ (Table 4).***

*Table 4. Associations of standardized ppFEV_1_ with VO_2peak_ (ml/kg/min) in total cohort and stratified by emphysema (E) and non-emphysema (NE) COPD phenotypes.*

|  |  | ***Model 1^a^*** | | ***Model 2^b^*** | | ***Model 3^c^*** | | ***Model 4^d^*** | |
| --- | --- | --- | --- | --- | --- | --- | --- | --- | --- |
|  | ***n*** | ***β*** | ***95% CI*** | ***β*** | ***95% CI*** | ***β*** | ***95% CI*** | ***β*** | ***95% CI*** |
| ***Total^e^*** | ***186*** | ***2.5*** | ***1.6, 3.3*** | ***2.6*** | ***1.8, 3.3*** | ***2.7*** | ***2.0, 3.3*** | ***2.5*** | ***1.8, 3.2*** |
| ***E-COPD*** | ***101*** | ***2.4*** | ***1.4, 3.3*** | ***2.5*** | ***1.7, 3.3*** | ***2.7*** | ***1.9, 3.5*** | ***2.7*** | ***1.8, 3.6*** |
| ***NE-COPD*** | ***85*** | ***2.6*** | ***0.9, 4.3*** | ***2.6*** | ***1.2, 4.0*** | ***2.0*** | ***0.7, 3.3*** | ***1.6*** | ***0.3, 2.9*** |

*ppFEV_1_ – percent of predicted forced expiratory lung volum in one second, VO_2peak_ – peak oxygen uptake, COPD – chronic obstructive pulmonary disease, β – regression coefficient, CI – confidence interval.*

*^a^Crude association. ^b^Adjusted for sex and age (years). ^c^Adjusted for sex, age, body mass index (BMI) and smoking status (former, current). ^d^Adjusted for sex, age, BMI, smoking status, systemic beta-blocker (yes, no) and inhaled bronchodilator (yes, no). ^e^Adjusted for COPD phenotype in all total sample models.*

***Stratified by COPD phenotype, one unit reduction in standardized ppFEV_1_ was, on average, associated with 2.7 (95% CI 1.8, 3.6) and 1.6 (95% CI 0.3, 2.9) ml/kg/min lower VO_2peak_ in E-COPD and NE-COPD, respectively after adjustment for sex, age, BMI, smoking status, beta-blocker and bronchodilator treatment (Table 4). The association between standardized ppFEV_1_ and VO_2peak_ did not differ statistically by phenotype (p-value for interaction = 0.220).”***

**Results, page 16, line 275-280:**

***“Phenotype related differences in VO_2peak_ between FEV_1Z-score_ and ppFEV_1_.***

***Adjusted for sex, age, BMI, smoking status, beta-blocker and bronchodilator treatment, VO_2peak_ was, on average, 2.2 (95% CI 0.8, 3.6) and 2.1 (95% CI 0.8, 3.5) ml/kg/min lower in E-COPD than in NE-COPD, when comparisons were made at similar FEV_1Z-score_ and ppFEV_1_, respectively.”***

**Discussion, page 17, line 294-299:**

***“In this study we found FEV_1Z-score_ to be positively associated with VO_2peak_. This association was stronger in E-COPD than in NE-COPD but did not differ statistically by phenotype. In corresponding analyses with standardized ppFEV_1_ similar estimates were observed. The exercise capacity was lower in E-COPD than in NE-COPD, but the phenotype related differences in VO_2peak_ were similar when comparisons were made using FEV_1Z-score_ and ppFEV_1_.”***

**Discussion, page 17, line 309-313:**

**“Although, we support the use of Z-scores of dynamic lung volumes in the diagnostic process in COPD, the associations of FEV_1_ with VO_2peak_ were similar using FEV_1Z-score_ and standardized ppFEV_1_. Thus we have no reason to state that Z-scores of FEV_1_ are preferable over percent of predicted values regarding the association with VO_2peak_ in COPD.*”***

**Discussion, page 18, line 325-329:**

***“The estimated difference in VO_2peak_ comparing E-COPD and NE-COPD was 2.2 (95% CI 0.8, 3.6) and 2.1 (95% CI 0.8, 3.5) ml/kg/min when patients were compared at similar FEV_1Z-score_ and ppFEV_1_, respectively. Thus, the difference in exercise capacity comparing these two COPD phenotypes seems independent on whether or not sex, age and height related variance in FEV_1_ is taken into account.”***

**Conclusions, page 22, line 413-415:**

***“Both the association of FEV_1_ with VO_2peak_ and the difference in VO_2peak_ comparing COPD phenotypes seems independent of sex, age and height related variance in FEV_1_.”***

**Abstract, page 3, line 46-49 and line 51-53:**

***“Similar estimates were obtained in analyses with standardized ppFEV_1_. Compared to NE-COPD, VO_2peak_ was 2.2 (95% CI 0.8, 3.6) and 2.1 (95% CI 0.8, 3.5) ml/kg/min lower in E-COPD, when adjusted for FEV_1Z-score_ and ppFEV_1_, respectively.”***

***“Both the association of FEV_1_ with VO_2peak_ and the difference in VO_2peak_ comparing COPD phenotypes seems independent of sex, age and height related variance in FEV_1_.”***

Reviewer #1: In table 1 and 2, there are no significant differences, is it correct? Apparently the groups are very different.

**Response:**

**In the original manuscript several variables presented in table 1 and 2 were tested between groups:**

**Methods, page 8, line 154-155:**

***“Lung function and exercise variables were compared between patients with E-COPD and NE-COPD using independent samples t-tests.”***

**The crude unadjusted difference in the following variables were reported with point estimates and 95% CIs. We have now added p-values in the revised version of the manuscript:**

**Results, page 11, line 204-209:**

***“Compared to patients with NE-COPD, patients with E-COPD had lower FEV_1Z-score_ (difference 0.69, 95% CI 0.36, 1.02, p < 0.001), lower FVC_Z-score_ (difference 0.42, 95% CI 0.05, 0.79, p = 0.028), lower FEV_1_/FVC_Z-score_ (difference 0.62, 95% CI 0.38, 0.86, p < 0.001), lower KCO_Z-score_ (difference 1.74, 95% CI 1.40, 2.07, p < 0.001), lower VA_Z-score_ (difference 0.91, 95% CI 0.56, 1.27, p < 0.001) and, by definition, lower T_L_CO_Z-score_ (difference 2.41, 95% CI 2.14, 2.68, p < 0.001) (Table 1).”***

**Results, page 13, line 226-230:**

***“Compared to patients with NE-COPD, patients with E-COPD had lower VO_2peak_ (difference 4.8 ml/kg/min, 95% CI 3.0, 6.5, p < 0.001), lower RER_peak_ (difference 0.04 95% CI 0.004, 0.08, p = 0.031), higher HRR (difference 6.1%, 95% CI 2.5, 9.6, p = 0.001), lower VE_peak_ (difference 14.8 l/min, 95% CI 7.8, 21.9, p < 0.001), lower VR (difference 5.5%, 95% CI -0.5, 11.5, p = 0.073) and lower S_p_O_2min_ (difference 1.7%, 95% CI 0.9, 2.6, p < 0.001) (Table 2).”***

**The implication of these differences are addressed as follows:**

**Discussion, page 19, line 341-346:**

***“We found both lower VR, suggesting higher ventilatory demand at peak exercise relative to estimated maximal ventilatory capacity, and a higher proportion reporting test termination due to dyspnea in E-COPD compared to NE-COPD. Mechanical ventilatory limitation may therefore be a principal pathophysiological mechanism explaining the difference in exercise capacity between patients with E-COPD and NE-COPD found in our study.”***

**Discussion, page 19, line 361-365:**

***“we cannot exclude the possibility that, additionally to being a marker of ventilatory capacity, any reduction in FEV_1_ may either impact or reflect gas exchange, ventilaton (V)/perfusion (Q) heterogeneity and ventilatory efficiency differently depending on COPD phenotype. Interestingly, but not confirmative of such speculation, we did find lower S_p_O_2min_ in E-COPD compared to NE-COPD.”***

**Discussion, page 20, line 376-380:**

***“the finding of both lower FEV_1Z-score_ and VO_2peak_ in patients with E-COPD, coincide with COPD phenotype being a positive confounder of the association between these key variables. We suggest that differentiation between T_L_CO below and above LLN should complement measures of dynamic lung volumes in future studies of exercise capacity in COPD.”***
