# Supplementary material for: Lung function and peak oxygen uptake in chronic obstructive pulmonary disease phenotypes with and without emphysema
Source: PLoS One. 2021 May 27;16(5):e0252386. doi: 10.1371/journal.pone.0252386 (PMC8158861; doi:10.1371/journal.pone.0252386)
Supplement: Attachment — Submitted filename: Response to Reviewers.docx [file pone.0252386.s003.docx]

Journal Requirements:

Please review your reference list to ensure that it is complete and correct. If you have cited papers that have been retracted, please include the rationale for doing so in the manuscript text, or remove these references and replace them with relevant current references. Any changes to the reference list should be mentioned in the rebuttal letter that accompanies your revised manuscript. If you need to cite a retracted article, indicate the article’s retracted status in the References list and also include a citation and full reference for the retraction notice.

**Response:**

**The reference list has been reviewed and found to be complete and correct. No retracted papers have been cited. There are no changes to the reference list.**

Additional Editor Comments (if provided):

Please, find the final comments of the reviewer and submit the manuscript to an English writer to improve the quality of the language

**Response:**

**With specific intention to improve the quality of the language as requested, the current version of the manuscript has been reviewed and revised by Ben M. Brumpton who is a native speaker of the English language.**

[Note: HTML markup is below. Please do not edit.]

Reviewers' comments:

Reviewer's Responses to Questions

**Comments to the Author**

1. If the authors have adequately addressed your comments raised in a previous round of review and you feel that this manuscript is now acceptable for publication, you may indicate that here to bypass the “Comments to the Author” section, enter your conflict of interest statement in the “Confidential to Editor” section, and submit your "Accept" recommendation.

Reviewer #1: All comments have been addressed

2. Is the manuscript technically sound, and do the data support the conclusions?

The manuscript must describe a technically sound piece of scientific research with data that supports the conclusions. Experiments must have been conducted rigorously, with appropriate controls, replication, and sample sizes. The conclusions must be drawn appropriately based on the data presented.

Reviewer #1: Yes

3. Has the statistical analysis been performed appropriately and rigorously?

Reviewer #1: Yes

4. Have the authors made all data underlying the findings in their manuscript fully available?

The [PLOS Data policy](http://www.plosone.org/static/policies.action#sharing) requires authors to make all data underlying the findings described in their manuscript fully available without restriction, with rare exception (please refer to the Data Availability Statement in the manuscript PDF file). The data should be provided as part of the manuscript or its supporting information, or deposited to a public repository. For example, in addition to summary statistics, the data points behind means, medians and variance measures should be available. If there are restrictions on publicly sharing data—e.g. participant privacy or use of data from a third party—those must be specified.

Reviewer #1: Yes

5. Is the manuscript presented in an intelligible fashion and written in standard English?

PLOS ONE does not copyedit accepted manuscripts, so the language in submitted articles must be clear, correct, and unambiguous. Any typographical or grammatical errors should be corrected at revision, so please note any specific errors here.

Reviewer #1: Yes

6. Review Comments to the Author

Please use the space provided to explain your answers to the questions above. You may also include additional comments for the author, including concerns about dual publication, research ethics, or publication ethics. (Please upload your review as an attachment if it exceeds 20,000 characters)

Reviewer #1: The authors modified the paper according to the indications.

They should put the significances on the tables (table 1 and 2), because the two

populations look different * < 0.05 or ** < 0.01

**Response:**

**As requested by the reviewer, the p-values comparing selected continuous variables between E-COPD and NE-COPD have been included in tables 1 and 2.**

Line 309 is in BOLD, please change it.

**Response:**

**Corrected.**
